# Quantitative and qualitative analysis of individual experiences post botulinum toxin injection ‐ United Kingdom Survey

**DOI:** 10.1002/ski2.265

**Published:** 2023-07-03

**Authors:** David Zargaran, Alexander Zargaran, Sara Sousi, Dawn Knight, Hannah Cook, Alexander Woollard, Julie Davies, Tim Weyrich, Afshin Mosahebi

**Affiliations:** ^1^ Department of Plastic Surgery University College London London UK; ^2^ British Association of Aesthetic Plastic Surgeons (BAAPS) Academy London UK; ^3^ Independent Patient Safety Advocate London UK; ^4^ Cosmetic Practice Standards Authority (CPSA) London UK; ^5^ UCL Global Business School for Health University College London London UK; ^6^ Department of Computer Science University College London London UK; ^7^ Friedrich‐Alexander University (FAU) Erlangen‐Nürnberg Erlangen Germany

## Abstract

**Introduction:**

In the United Kingdom (UK), complications that arise following the administration of Botulinum Toxin are reported to the Medicines and Health Regulatory Agency (MHRA) via the Yellow Card Reporting Scheme. Over the past decade, there has been a significant increase in the number of non‐surgical aesthetic procedures. Concerns have been raised that the MHRA is not fully capturing complications in terms of volume and impact on patients.

**Aim:**

This novel study explores the lived experiences of individuals who have experienced an adverse event following administration of Botulinum Toxin for aesthetic purposes. Using a combination of qualitative and quantitative methodologies, this analysis evaluates data relating to long‐lasting physical, psychological, emotional, and financial sequelae of complications arising from cosmetic Botulinum Toxin injections in the UK.

**Methods:**

A mixed method, qualitative and quantitative approach was adopted to gain comprehensive insights into patients' experiences. A focus group which comprised patient representatives, psychologists, and researchers reached a consensus on a 17‐question survey which was disseminated via social media channels. Deductive thematic analysis was used to analyse coded themes. Furthermore, for secondary analysis, sentiment analysis was used computationally as an innovative approach to identify and categorise free text responses associated with sentiments using natural language processing (NLP).

**Results:**

In the study, 655 responses were received, with 287 (44%) of respondents completing all questions. The mean age of respondents was 42.6 years old. 94.1% of respondents identified as female. In the sample, 79% of respondents reported an adverse event following their procedure, with the most common event being reported as ‘anxiety’. Findings revealed that 69% of respondents reported long‐lasting adverse effects. From the responses, 68.4% reported not having recovered physically, 63.5% of respondents stated that they had not recovered emotionally from complications, and 61.7% said that they have not recovered psychologically. In addition, 84% of respondents stated that they do not know who regulates the aesthetics industry. Furthermore, 92% of participants reported that their clinic or practitioner did not inform them about the Yellow Card Reporting Scheme. The sentiment analysis using the AFINN Lexicon yielded adjusted scores ranging from −3 to +2, with a mean value of −1.58.

**Conclusion:**

This is the largest survey in the UK completed by patients who experienced an adverse outcome following the aesthetic administration of Botulinum Toxin. Our study highlights the extent of the challenges faced by patients who experience an adverse event from physical, emotional, psychological, and financial perspectives. The lack of awareness of MHRA reporting structures and the lack of regulation within the UK's cosmetic injectables sector represent a significant public health challenge.

**What is already known?**

- This is the academic largest survey, to date, in the UK completed by patients who experienced an adverse outcome following the aesthetic administration of Botulinum Toxin.
- Our study highlights the extent of the challenges faced by patients who experience an adverse event from physical, emotional, psychological, and financial perspectives.
- The lack of awareness of MHRA reporting structures and the lack of regulation within the UK's cosmetic injectables sector represent a significant public health challenge.

**What does this study add?**

- This study presents a snapshot of the true impact on patients’ lives stemming from a lack of regulation. This is the largest survey completed by patients who experienced an adverse outcome following the aesthetic administration of Botulinum Toxin. The findings contribute a valuable foundation towards greater understanding of the cosmetic injectables industry, with a view to facilitating appropriate regulation.

## 1 INTRODUCTION

In the United Kingdom (UK), complications that arise following the administration of Botulinum Toxin should be reported to the Medicines and Health Regulatory Agency (MHRA) via the Yellow Card Reporting Scheme.^1^ Over the past decade, there has been a significant increase in the number of non‐surgical aesthetic procedures performed.^2^ Within the UK, this follows from the 2013 publication of the Keogh report which highlighted concerns regarding the safety of the entire cosmetic injectables industry.^3^ Further, recent data have demonstrated concerns that existing methods of capturing complications may significantly underreport complications, leaving an untold public health burden.^4^, ^5^ Failure to understand the extent of the risk poses a significant public health challenge to regulators. The UK government has announced plans to introduce licencing schemes to ‘prevent vulnerable people from being exploited’.^6^

The rapid growth in this sector increases the potential risk of adverse outcomes. The MHRA has responded by increasing the accessibility of the Yellow Card scheme to facilitate provisions via the Yellow Card Website or App.

When considering how users interact with and adopt a technology, the Technology Acceptance Model (TAM) Theory described by Davis in 1989, is considered the seminal information systems theory model to understand the adoption of new technology.^7^ TAM considers two key elements as critical before a user adopts a new technology: perceived usefulness and perceived ease of use. Elements relating to ease of use include accessibility and the “degree to which a person believes that using a particular system would be free from effort”. Making the UK's Yellow Card Scheme available via a website and App would increase the perceived ease of use. However, when considering usefulness, one must consider the content of information provided and whether users believe that reporting would make a real difference.

In terms of “usefulness”, individuals who access the Yellow Card site see four key sections.

1. Whose side effect (demographic details)
2. Side effects (details relating to the adverse event experienced)
3. About the medicines suspected to have caused a side effect (details relating to batch number, lot number, indication, start date, stop date and method of administration etc.)
4. Additional details (information relating to other medicines, taken alongside the medicine suspected to have caused the reaction, and medical history etc.)

The clear focus of these questions centres around the medical impact of a suspected adverse reaction. The Nuffield Council on Bioethics publication on the ethical issues of Cosmetic Procedures warned that the growth in this market must be carefully monitored and supported to ensure ethical practice.^8^ Part of this ethical practice is ensuring the bio‐, psycho‐ and social elements of practice are considered and monitored. This is particularly important in considering the impact of Body Dysmorphic Disorder (BDD) which is a significant risk factor in aesthetic procedures.^9^ There is also an important financial consideration to be aware of in this sector, adding further complexity to the industry. With these considerations, to fully understand the impact of any medicine, including Botulinum Toxin, a broader scope of questioning may be considered.

Empowering patients to report their own adverse events has already been shown to yield a higher number of reported adverse drug reactions and more detailed responses in their reports. Generating new potential signals is critical in pharmacovigilance and improve our understanding of the impact of adverse events on patients' lives.^10^

## 2 AIM

The study in this paper explores the lived experiences of individuals who experienced an adverse event after they were administered Botulinum Toxin for aesthetic purposes. Using both qualitative and quantitative methodologies, the analysis identifies data based on long‐lasting physical, psychological, emotional and financial sequelae of Botulinum Toxin related adverse events. Other factors including redress and support are also explored.

## 3 METHODS

A concurrent mixed methods qualitative and quantitative approach^11^ was adopted to address the research aims of gaining a comprehensive understanding of patients' experiences. An oversight board formed by members of the Joint Council for Cosmetic Practitioners (JCCP), the British Association of Aesthetic Practitioners (BAAPS), the Cosmetic Practice Standards Authority (CPSA) and University College London (UCL) developed and distributed the survey.

Representatives from each group formed a focus group which comprised patient representatives, psychologists, researchers, and leadership of the organisations outlined above. The group designed a 17‐question survey with elements capturing quantitative and qualitative responses (Appendix 1).

### 3.1 Distribution

An advert was created and distributed across the social media channels of the JCCP, the British Beauty Council (BBC), the Hair and Beauty Industry Authority (HABIA), primarily across Facebook, Twitter and LinkedIn channels. Individuals were invited to self‐identify in response to the advert. Individuals who had previously undergone Botulinum Toxin injections for cosmetic purposes, aged over 18 and under 75 were deemed as eligible to participate. Participants were invited to complete the survey between 1 January 2023 and 31 March 2023 and data included over the 3‐month window was included for further analysis. Data were collected using Qualtrics (Qualtrics International, Seattle, Washington, USA).

### 3.2 Qualitative analysis

Deductive thematic analysis was used to analyse coded themes. Furthermore, as a secondary analysis, sentiment analysis was performed to computationally identify and categorise free text responses and associate with a sentiment using natural language processing (NLP).^12^ Participants' free text entries were processed and filtered using R software v 4.0.3, and the R package tidytext (version 0.4.1) was used to undertake the analysis. Three lexicons were cross referenced, namely AFINN,^13^ Bing^14^ and nrc.^15^ The purpose was to create a surrogate marker for the impact of adverse events on patients.

### 3.3 Ethical approval

The University College London Research Ethics Committee (UCL REC) reviewed and approved this study, approval ID: 24 379.001. Participants received a link to a participant information leaflet (PIL). On‐line informed consent was obtained from all participants in the survey cohort.

## 4 RESULTS

In total, 655 responses were received with 287 (44%) of respondents completing all questions. 143 (22%) of respondents did not answer any questions and these were excluded from the analysis, resulting in a sample size of 511.

**FIGURE 1:**
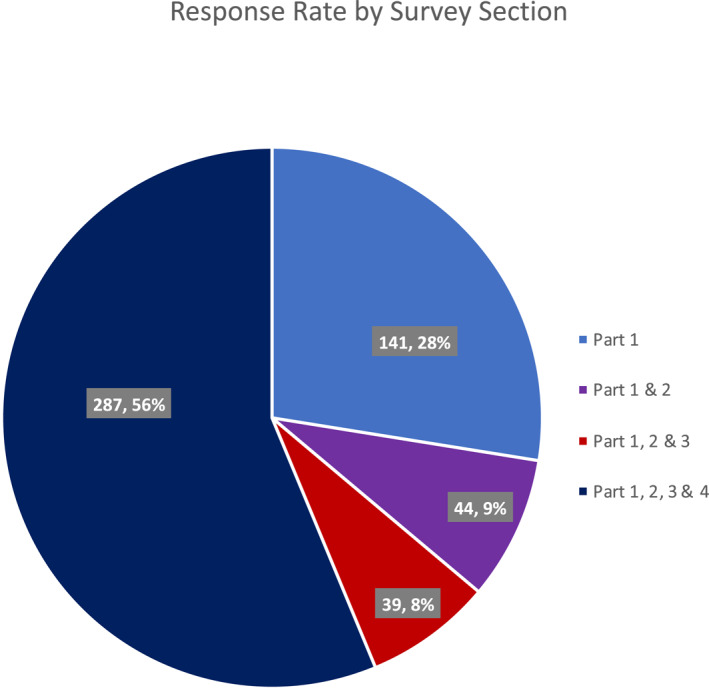
Response rate by survey section.

The survey was divided into four parts: (1) Demographic data, (2) Understanding your experience, (3) Long term consequences, and (4) Redress. In order to progress to the next part, participants needed to complete all the questions for each section. Only 141 individuals responded to part 1, 44 to parts 1 and 2, 39 to parts 1, 2 and 3, and 287 to all four parts of the survey (Figure 1).

### 4.1 Demographics

**FIGURE 2:**
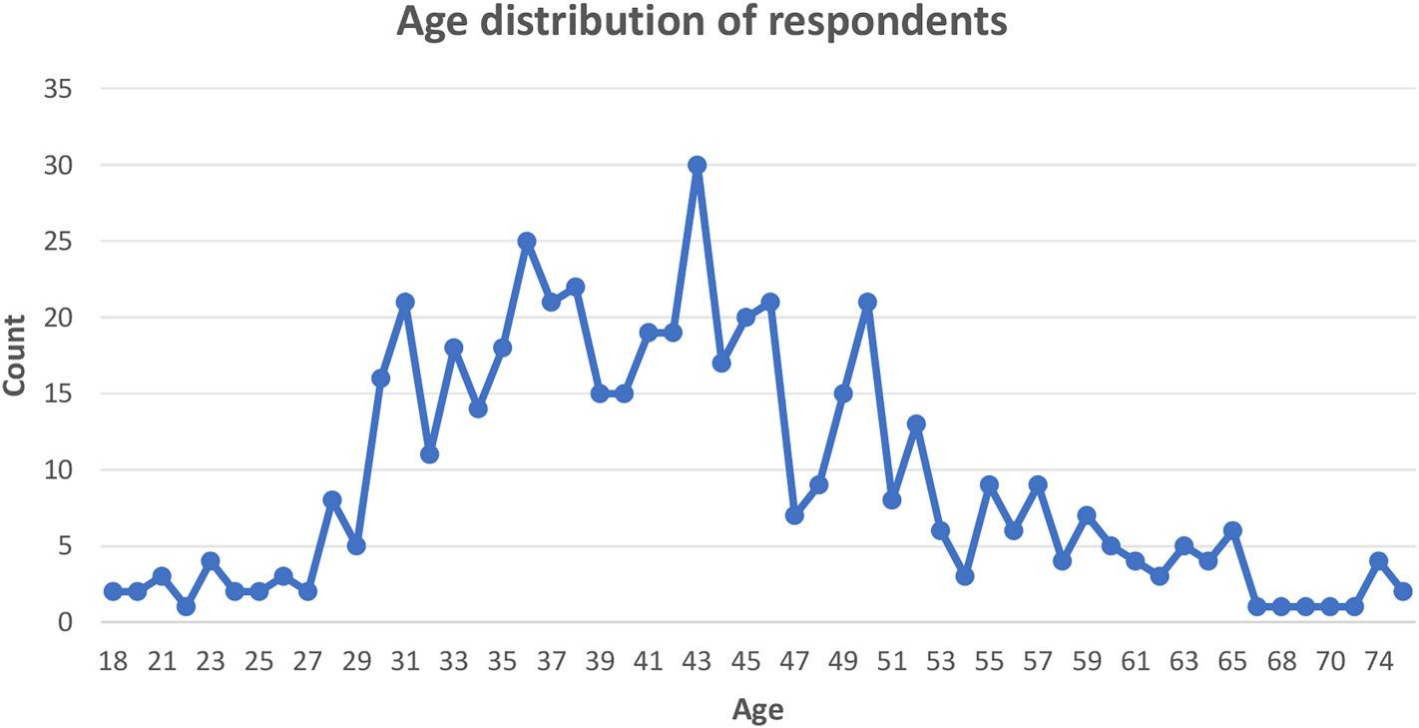
Age distribution by respondents.

The mean age of respondents 42.6 (Figure 2). Most respondents identified as female (*N* = 481, 94.1%). In the sample, there were 24 (4.7%) male respondents. The remaining respondents in the sample identified as non‐binary (*N* = 4, 0.8%) or other (*N* = 2, 0.4%). Ethnicity data of participants was classified according to the 2021 UK census ethnicities. Most respondents identified as white (*N* = 341, 84.1%), followed by Black, Black British, Caribbean or African (*N* = 42, 8.2%), then Other (*N* = 30, 5.9%), and Asian or Asian British (*N* = 9, 1.8%) (Table 1).

**TABLE 1:** Representation of survey participant ethnicities.

Ethnicity	N	%
White	430	84.1%
English, Welsh, scottish, Northern Irish or British	225	44.0%
Any other white background	183	35.8%
Irish	19	3.7%
Roma	3	0.6%
Black, black british, Caribbean, or African	42	8.2%
Mixed or multiple ethnic groups	14	2.7%
Any other mixed or multiple ethnic background	12	2.3%
White and Asian	8	1.6%
Caribbean	5	1.0%
White and black African	1	0.2%
White and black Caribbean	1	0.2%
Any other black, black british, or Caribbean background	1	0.2%
Other ethnic group	30	5.9%
Any other ethnic group	26	5.1%
Arab	4	0.8%
Asian or Asian british	9	1.8%
Any other Asian background	5	1.0%
Indian	2	0.4%
Chinese	2	0.4%
Total	511	100.0%

In the survey, 79% of respondents reported an adverse event following their procedure, whilst 76 (21%) out of the 368 respondents reported that they had no unexpected problems and/or difficulties. Table 2 outlines the adverse events experienced by individuals with *n* denoting the frequency with which respondents reported an issue.

**TABLE 2:** Problems experienced by survey participants.

Problem/Adverse Effect	N
Pain	83
Anxiety	85
Panic attacks	46
Depression/Low mood	22
Headache/migraine	75
Brain fog	33
Tinnitus/ear ringing	31
Twitching	18
Dizziness	33
Diarrhoea	12
Weight loss	17
PoTS	6
MCAS	7
Autoimmune	7
Histamine/allergy	24
Electrical/nerve zaps	14
Bladder/UTI/urination	24
Bruising	12

### 4.2 Sentiment analysis

#### 4.2.1 Bing lexicon

**FIGURE 3:**
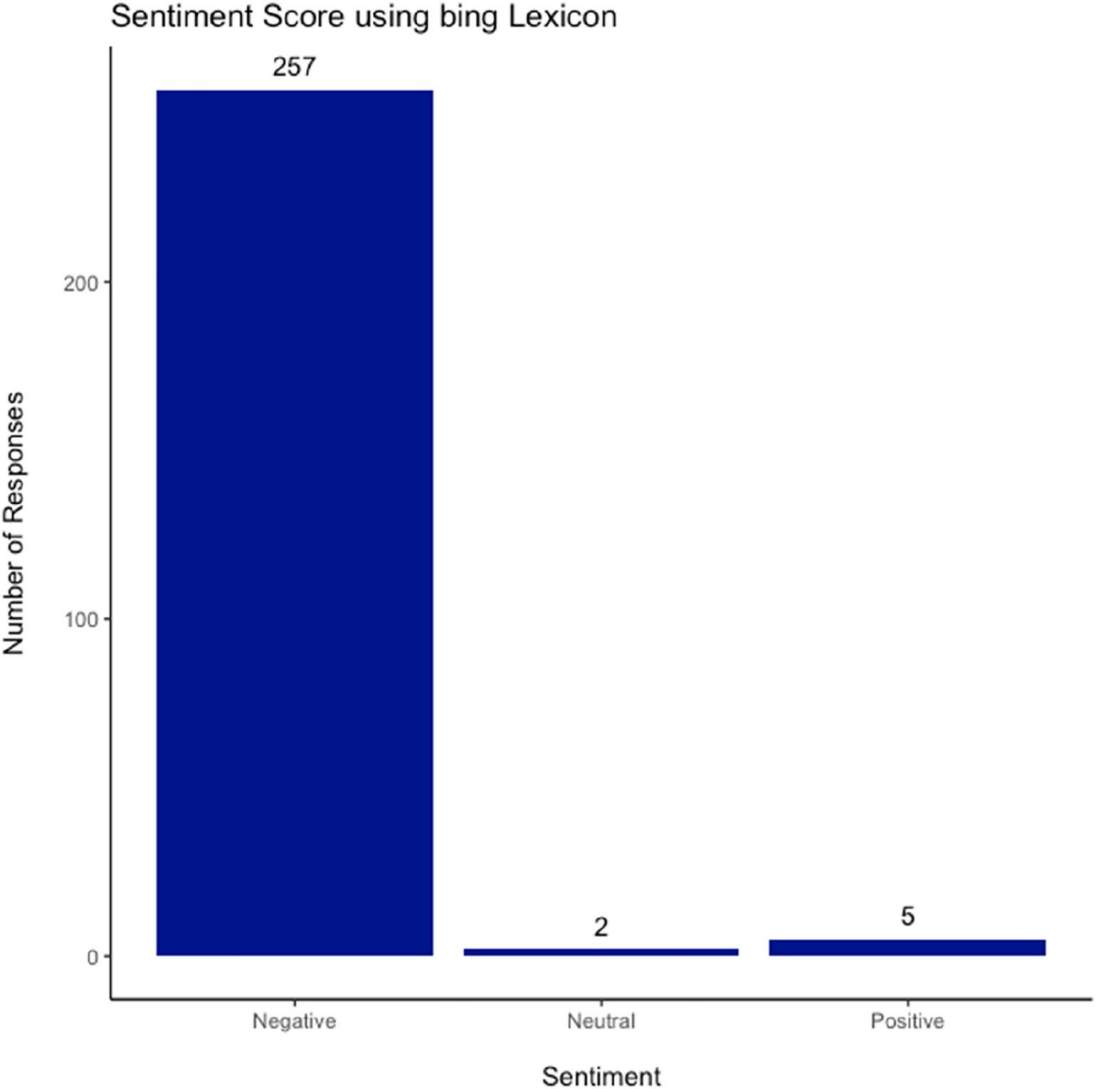
Sentiment Score using bing Lexicon.

Using the Bing lexicon (Figure 3), the words were categorised into either negative or positive. Post‐mapping 263 responses were given a sentiment score (StSc). The mean StSc was −0.89, it ranged from −1 to +1 with only five responses being overall positive (StSc >0), and two neutral (StSc = 0). The words from each response were assigned either a positive or negative sentiment based on the lexicon, the words that had zero sentiment were removed. Then for each response the number of negative words was subtracted from the number of positive words and divided by the total number of words with an assigned sentiment.

#### 4.2.2 AFINN dictionary

**FIGURE 4:**
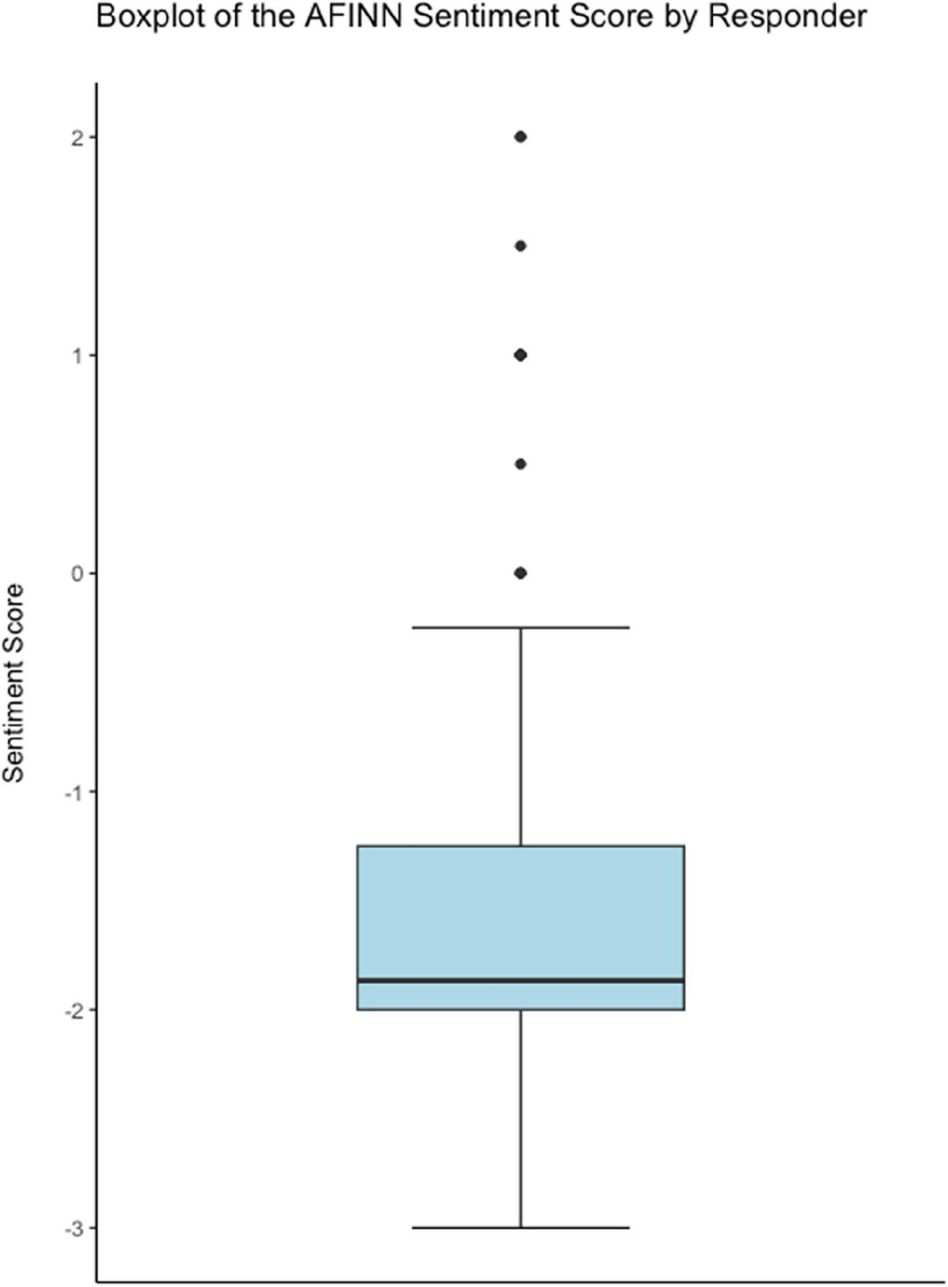
Boxplot of the AFINN sentiment score by responder.

Using the AFINN lexicon, the words were given a score ranging from −5 to +5; these were summed for each response and divided by the number of words in each response to give an adjusted sentiment score due to heterogeneity of length of responses. The words that did not have a value assigned based on the lexicon were excluded. The adjusted scores ranged from −3 to +2, with a mean value of −1.58 (Figure 4).

#### 4.2.3 nrc Dictionary

**FIGURE 5:**
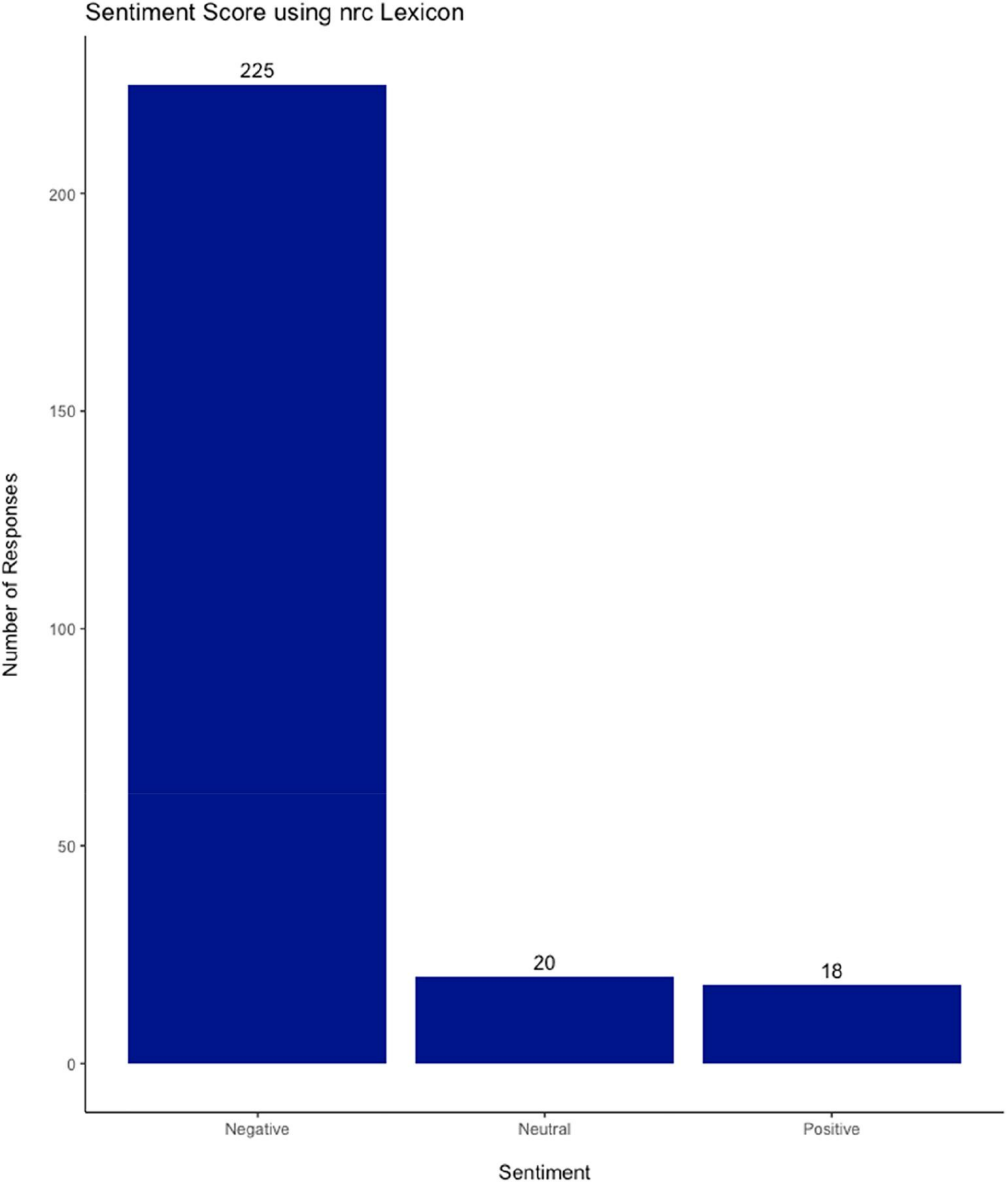
Sentiment Score using nrc Lexicon.

Using the nrc lexicon, the words were categorised into positive, negative, anger, anticipation, disgust, fear, joy, sadness, surprise, and trust. For the positive and negative analysis, the same methodology was followed to obtain the sentiment score (StSc). The mean StSc was −0.55. It ranged from −1 to +1 with 18 responses being overall positive (StSc >0), and 20 neutral (StSc = 0) – Figure 5.

#### 4.2.4 Administration data

Most administrators (Table 3) were health care professionals (80.9%). A doctor administered Botulinum Toxin in 145 (39.2%) cases, a nurse in 135 (36.5%), a dentist in 17 (4.6%), and a pharmacist in three (0.8%) cases. A beautician administered Botulinum Toxin for 51 (13.8%) respondents and the remaining were either classified as Other in 13 (3.5%) cases or unknown for six (1.6%) responses.

**TABLE 3:** Administrators of procedure reported by survey participants.

Administrator	N	%
Doctor	145	39.2%
Nurse	135	36.5%
Beautician	51	13.8%
Dentist	17	4.6%
Other	13	3.5%
Don't know/Can't be sure	6	1.6%
Pharmacist	3	0.8%
Total	371	100%

**FIGURE 6:**
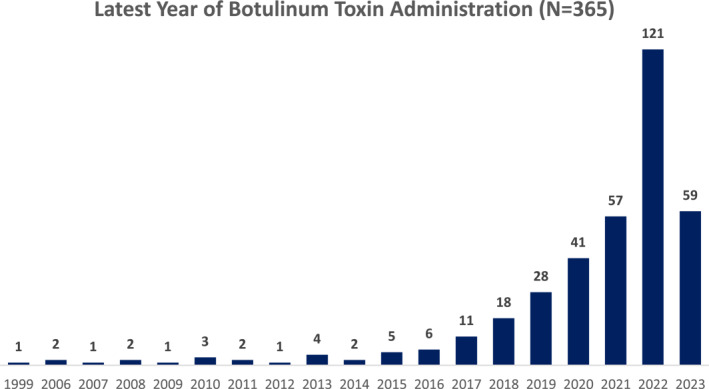
Latest year of Botulinum Toxin administration (*N* = 365).

The most common administration year was 2022 with 121 (33.2%) cases, followed by 2023 at 59 (16.2%) and 2022 at 57 (15.8%) (Figure 6).

Table 4 lists where Botuliunum Toxin was administered.

**TABLE 4:** Location of procedure.

Location	N	%
Clinic	216	58.4%
Beauty salon	46	12.4%
Spa	38	10.3%
Other	36	9.7%
Doctor's office	10	2.7%
No mention	6	1.6%
Hospital	6	1.6%
Neurologist's office	3	0.8%
Plastic surgeon's office	3	0.8%
Dermatologist's office	2	0.5%
Office	1	0.3%
Walmart	1	0.3%
Individual suite	1	0.3%
Work	1	0.3%
Laser clinic	1	0.3%
Medical facilities	1	0.3%
Home	22	5.9%
Dental practice	12	3.2%
Total	371	100.0%

#### 4.2.5 Long term consequences

In the survey, 69% of participants reported long‐term consequences following their adverse experience (Table 5). Of those who experienced physical harm, injury or illness, 223 out of 326 respondents (68.4%) reported not having fully recovered. In the sample, 63.5% reported not having recovered emotionally. Further, 61.7% reported not having recovered psychologically.

**TABLE 5:** Long term reported effects from survey participants.

Have there been any long‐lasting effects following your procedure?	N	%
No	101	31.0%
Yes	225	69.0%
Total	326	100.0%

#### 4.2.6 Financial costs

**FIGURE 7:**
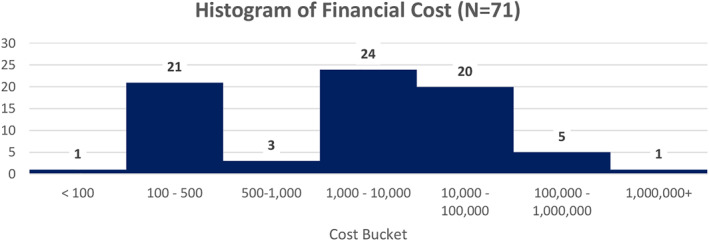
Histogram of financial cost (*N* = 71).

**FIGURE 8:**
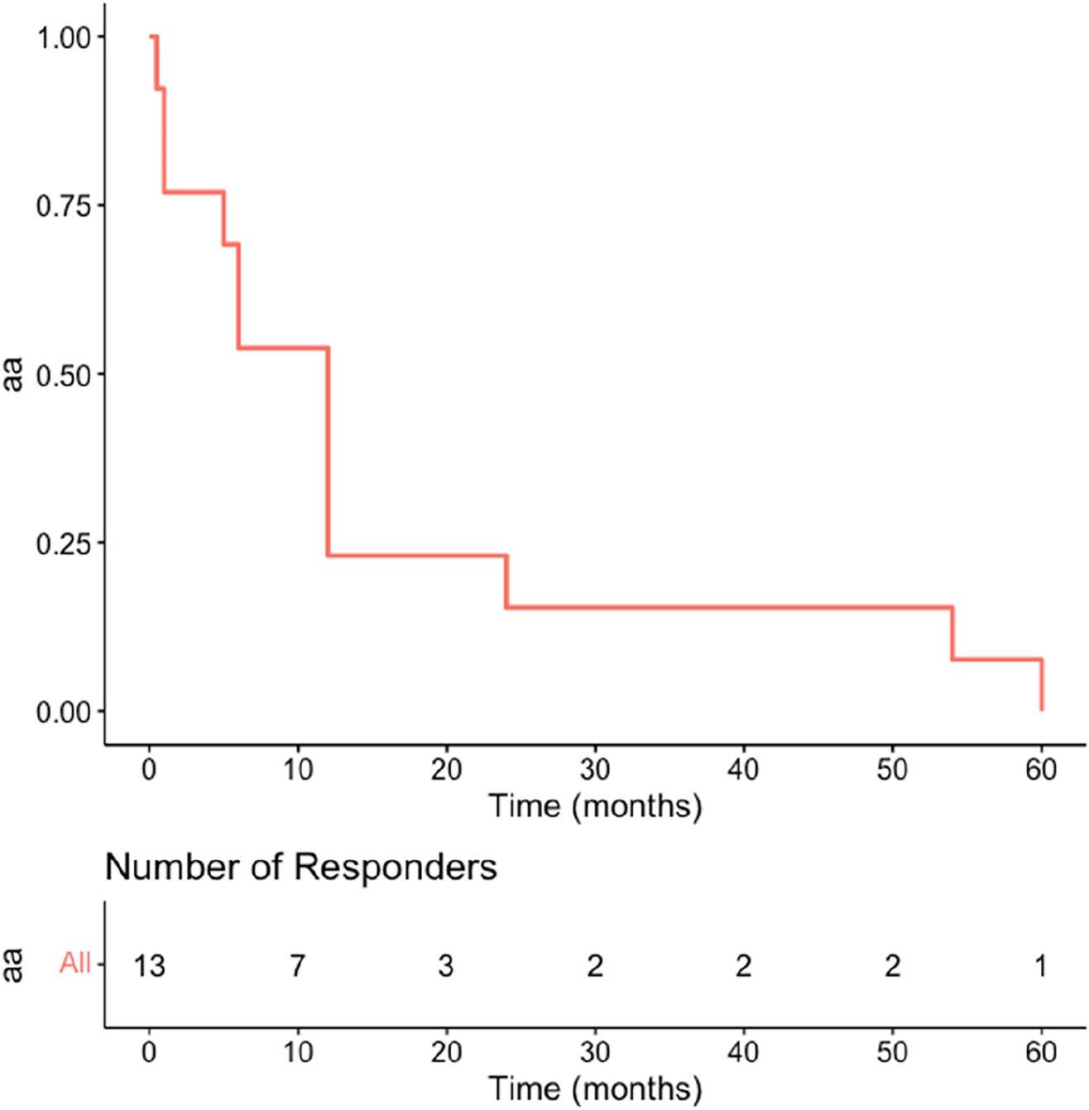
Time to return to work.

Respondents to this question reported costs either in terms of monetary values or time they took off work. Figure 7 lists costs incurred. Time off work is illustrated using a Kaplan Meier chart with time to event being the return to work (Figure 8).

#### 4.2.7 Redress

In the study, 78.4% (225 amongst 287) of practitioners refused to support their patients when there were complications following procedures. A further 16.3% of respondents reported that their practitioner advised them to visit an accident and emergency department after they experienced a complication. Findings reveal that 54.7% of respondents reported seeking help from alternative channels via the NHS (National Health Service). Those who did not seek help from other channels outlined their reasons (see Table 6).

**TABLE 6:** Reported reasons for not seeking help.

Reasons for not seeking help	N
No need	22
Wouldn't believe me	12
Non‐UK	9
Improved with time	9
N/A	6
Support group	4
Did not know	3
Unaware	3
Symptoms not severe enough	2
Nothing could be done	3
Did not think to	2
Told would pass with time	2
Didn't trust they would help	1
Didn't know how	1
Said could not be by botulinum Toxin	1
Embarrassed	2
Doctor should have taken responsibility	1
Followed the given guidance	1
Support existed from nurses	1
Because most doctors said nothing was wrong	1
Told me it was fine	1
Lack of knowledge	2
Lack of trust	1
Nothing done	1
Because nobody believes that botulinum Toxin has side effects	1
Other treatment received from pharmacy	1
NHS not responsible for private work	1
Stopped having bou	1
Unable	1
Did not think it was due to botulinum Toxin	1
Assumed would improve naturally	1
Told it would fade with time	1
Told symptoms were normal	2
Did not realise symptoms were due to the botulinum Toxin	1
Because the system isn't designed that way	1
Alternative medicine route	1
No proof that botulinum Toxin caused this	1
Unsure	1
Administrator stated botulinum Toxin does not work always hence results not being as expected	1
No chance to do so	1
No help would be offered	1

In the survey, 84% (241) of the respondents stated that they do not know who regulates the aesthetics industry. The responses of those who reported knowing who regulated the industry are presented as free text in Table 7. The most frequent response (35%) was the Food and Drug Administration (FDA) despite being a UK study, and second ‘no‐one’ (provided in 26%) 92% (264/287) of participants reported not being told by their clinic or practitioner about the yellow card reporting scheme.

**TABLE 7:** Participant perceived regulator of aesthetics industry.

Regulator Responses	N	%
FDA	15	32.6%
No one	10	21.7%
Null	3	6.5%
CPSA	2	4.3%
JCCP	2	4.3%
GMC	2	4.3%
Big pharmaceutical companies	1	2.2%
DHSC	1	2.2%
No one, possibly FDA	1	2.2%
FDA, CDC	1	2.2%
MHRA	1	2.2%
Allergan*	1	2.2%
No one, clinic had RQIA (regulation and quality improvement Authority)	1	2.2%
Institute regulating medication each country	1	2.2%
State board	1	2.2%
CDC	1	2.2%
California disease Control	1	2.2%
JCCP, british beauty Council	1	2.2%
Total	46	100%

## 5 DISCUSSION

This is the largest survey completed by patients who experienced an adverse outcome following the aesthetic administration of Botulinum Toxin. The findings contribute a valuable foundation towards greater understanding of the cosmetic injectables industry, with a view to facilitating appropriate regulation. One of the most striking features of this study is the number of patients reporting an adverse outcome. Our sample included a total of 511 respondents, which when compared to the 188 adverse reports listed by the MHRA between 1991 and 2020, immediately suggests a significant under‐reporting of officially recognised adverse events within the UK.^5^ The notion of under‐reporting from complications of aesthetic Botulinum Toxin and the untold public health burden is consistent with the fact that 84% of study respondents were unaware who regulated the aesthetics industry in the UK. A further 92% of participants in our study reported not being told by their clinic or practitioner about the Yellow Card Reporting Scheme. Recognition and reporting of adverse events relating to drug administration are integral to informed consent processes. Our study suggests a lack of patient empowerment and education regarding avenues for redress.

There may also be a perceived lack of ‘usefulness’ in reporting according to Davis' TAM model. Table 6 offers an insight into why some individuals did not seek help. Certain themes emerged such as lack of trust, lack of awareness/knowledge, or concerns that their healthcare professional did not believe them. Of the patients (16%) who reported knowing who regulated the aesthetics industry in the UK, 35% reported the FDA and 26% reported ‘no one’. This is concerning and highlights that lack of regulation in this sector may be perpetuating patient concerns. The JCCP published their 10 point plan in 2021^16^ and this highlighted as Point 1 that there is “no primary legislation in the UK to regulate this sector, and with the exception of hair restoration surgery there are no restrictions set down to determine who can legally perform the more invasive procedures relating to chemical peels, lasers, injectables and dermal fillers.” This lack of legislation presents a potential risk to patients and the public. The move to introduce licencing in the UK is a step in the right direction, but how this will take shape will be important, and the findings of this study will also inform the extent of the challenge.^17^

This study also demonstrates the potentially long‐lasting effects of an adverse reaction to Botulinum Toxin. In the study, 79% of respondents reported experiencing an adverse event whilst 21% reported no adverse event. The most commonly reported adverse event which participants in the study noted was ‘anxiety’ (*n* = 85). Whilst causality cannot be inferred from this observational study, the reported anxiety or panic attacks (*n* = 46) which patients attributed to their Botulinum Toxin injections may represent an association between the administration of Botulinum Toxin and complications. These findings highlight the level of anxiety surrounding complications of this poorly regulated industry. Future research should examine whether such complications are a direct result of administered intervention or a side effect of concerns relating to how it is administered. Further research should also evaluate the extent and range of other reported adverse events (Table 2). The challenge with evaluating these data is that without further information relating to the specific product administered – such as the data captured in Section 3 of the MHRA Yellow Card Forms – causality would be impossible to determine. The presence of an association between various factors, however, can be helpful in understanding the extent of the challenge.

While 69% of respondents in the study reported long‐lasting effects, 68.4% reported not having recovered physically, 63.5% not having recovered emotionally and 61.7% said that they had not recovered psychologically. These findings demonstrate a key concern for participants who continue to experience injury and trauma following adverse reactions. Whilst causality may not be determined, the impact on patients' physical, emotional, and psychological recovery must addressed. Furthermore, Figures 7 and 8 demonstrate the financial costs associated with an adverse outcome. Costs range from the £1,000s through to one individual reporting costs over £1,000,000. Six individuals reported that they were physically disabled and handicapped following an adverse event.

Overall, this study presents a snapshot of the true impact on patients' lives stemming from a lack of regulation. Social media platforms such as Facebook have become a source for support and at the time of writing, “Botox Dysport (Side Effects) Support” Group has over 27 000 members, “Victims of Botched Surgery & Malpractice, Patient Support UK” 8400 members, “BOTCHED FILLERS & HYALURONIDASE DAMAGE SUPPORT GROUP” 5700 members. The size of the groups presents an important signal for legislators and those involved in health policy to evaluate whether existing mechanisms and systems provide appropriate support.

This study also leveraged sentiment analysis as a secondary outcome measure to evaluate participants' free text responses. By exploring three commonly used lexicons, that is, AFINN, Bing, nrc, we were able to evaluate and compare the impact of the complications on patients. The predominantly negative responses are consistent with the nature of the study. However, the AFINN lexicon and subsequent analyses provided an interesting insight into the extent of the negative emotions and sentiments. The mean value of −1.58 and range of +2 to −3 gave an interesting insight into the participant cohort. This form of artificial intelligence is likely to develop and enhance future qualitative analyses.

### 5.1 Limitations

As the design of this study is retrospective, the quality of the data is limited by recall bias,^18^ where we rely on respondents' recollections of events. A more robust methodology would be to collect data prospectively.^19^ MHRA could adopt this remit with access to significantly greater resources to address unsafe practices.

The design of an on‐line survey might have led to potential attrition bias.^20^ Of the 655 respondents, only 287 (44%) completed all the questions. Our focus group sought to mitigate this in the design of the study by balancing the length of the survey with the ambition to be as comprehensive as possible.

Further, through asking participants to self‐identify in response to an advertisement, the data are subject to population/respondent bias, and as such the data will not be representative of the entire population. However, our focus was to evaluate adverse events and as such the methodology selection is justified in providing a snapshot of the industry.

## 6 CONCLUSION

This is the largest survey completed by patients who experienced an adverse outcome following the aesthetic administration of Botulinum Toxin. Our study highlights the physical, emotional, psychological, and financial perspective challenges patients face when they experience complications. The lack of awareness of MHRA reporting structures is of particular concern. Coupled with the lack of regulation within the UK's cosmetic injectables sector, this presents a significant public health challenge. Finally, we call for further research and policy initiatives to raise awareness of patients' experiences and rights in this burgeoning industry.

## CONFLICT OF INTEREST STATEMENT

All authors have completed the Unified Competing Interest form (available on request from the corresponding author) and declare: no support from any other organisation for the submitted work; the research presented was sponsored by QUAD A – see below, no other relationships or activities that could appear to have influenced the submitted work.

## Author contribution

**David Zargaran**: Conceptualization (lead); Data curation (lead); Formal analysis (lead); Funding acquisition (lead); Investigation (lead); Methodology (lead); Project administration (lead); Resources (lead); Software (lead); Supervision (lead); Validation (lead); Visualization (lead); Writing – original draft (lead); Writing – review & editing (lead). **Alexander Zargaran**: Data curation (equal); Formal analysis (equal); Investigation (equal); Methodology (equal); Project administration (equal). **Sara Sousi**: Formal analysis (equal); Software (equal); Visualization (equal). **Dawn Knight**: Methodology (equal); Project administration (equal); Resources (equal). **Hannah Cook**: Methodology (equal). **Alexander Woollard**: Investigation (equal); Resources (equal). **Julie Davies**: Writing – review & editing (equal). **Tim Weyrich**: Conceptualization (equal); Visualization (equal). **Afshin Mosahebi**: Resources (equal); Software (equal); Supervision (equal). Validation (equal); Visualization (equal); Writing – original draft (equal); Writing – original draft (equal).

## ETHICS STATEMENT

Ethical approval was sought and granted by the UCL Research Ethics Committee.

## Supporting information

Supporting Information S1

## Data Availability

Data available on request from the authors.
