# Supplementary material for: Quantitative and qualitative analysis of individual experiences post botulinum toxin injection ‐ United Kingdom Survey
Source: Skin Health Dis. 2023 Jul 3;3(5):e265. doi: 10.1002/ski2.265 (PMC10549845; doi:10.1002/ski2.265)
Supplement: Supplementary file 1 — Supporting Information S1 [file SKI2-3-e265-s001.docx]

Appendix 1: Capturing individual experiences post botulinum toxin injection administration – survey questions

**Part 1: Demographic Data**

In order to understand the types of experiences you may have had, we would appreciate some anonymous data regarding your age and some equality data.

- 1. How old are you?
  2. What gender do you identify as?
     1. Male
     2. Female
     3. Non-binary
     4. Other
  3. What ethnicity do you identify as according to the following 2021 UK census ethnicities?

Asian or Asian British

Indian

Pakistani

Bangladeshi

Chinese

Any other Asian background

Black, Black British, Caribbean or African

Caribbean

African

Any other Black, Black British, or Caribbean background

Mixed or multiple ethnic groups

White and Black Caribbean

White and Black African

White and Asian

Any other Mixed or multiple ethnic background

White

English, Welsh, Scottish, Northern Irish or British

Irish

Gypsy or Irish Traveller

Roma

Any other White background

Other ethnic group

Arab

Any other ethnic group

**Part 2: Understanding your experience**

- 1. What unexpected problems / difficulties have you faced following your procedure – please share and describe your experiences in as much detail as possible ?
  2. Who administered your Botulinum Toxin (job role) – drop down
     1. Doctor
     2. Nurse
     3. Dentist
     4. Pharmacist
     5. Beautician
     6. Other
     7. Don’t know / Can’t be sure
  3. When was it administered? (Year)
  4. Where was it administered? (home, clinic, beauty salon)

**Part 3: Long term consequences**

- 1. Have there been any long-lasting effects following your procedure?

8b. If so, please explain:

- 1. If you suffered any physical harm, injury or illness, as a direct consequence of your treatment, have you now recovered physically?
  2. If you suffered any emotional impacts such as being upset, dissatisfied or worried as a direct consequence of your treatment, have you now recovered emotionally? ?
  3. If you suffered from any psychological impacts such as a deterioration in your mood, your thoughts, your outlook, or your mental health, have you now recovered psychologically?
  4. What have the financial implications of your treatment journey and recovery (so far) been?

**Part 4: Redress**

- 1. Did the clinic or practitioner agree to support you to set things right?
  2. Did the clinic or practitioner advise you to go to A+E for any complication that you might have experienced following your procedure?
  3. Did you seek assistance from other channels via the NHS? (GP, 111 service, or any another registered healthcare practitioner( e.g Nurse, Dr, GP, Dentist, Surgeon etc)
- If so, please detail your experience?
- If not, why not?
  1. Do you know who regulates the aesthetics industry?
     1. Yes
     2. No
        1. If yes – please state who?
  2. Did the clinic or practitioner tell you about the Yellow Card reporting scheme for side effects or complications
     1. Yes
     2. No
